# Targeted delivery of Grem1 and IL-10 separately by mesenchymal stem cells effectively mitigates SETD2-deficient inflammatory bowel disease

**DOI:** 10.7150/thno.105876

**Published:** 2025-01-13

**Authors:** Rebiguli Aji, Yue Xu, Ziyi Wang, Wenxin Feng, Liming Gui, Hanyu Rao, Wei Zhang, Ningyuan Liu, Wei-Qiang Gao, Li Li

**Affiliations:** 1 State Key Laboratory of Systems Medicine for Cancer, Renji-Med X Clinical Stem Cell Research Center, Ren Ji Hospital, School of Medicine and School of Biomedical Engineering, Shanghai Jiao Tong University, Shanghai, China.; 2 School of Biomedical Engineering and Med-X Research Institute, Shanghai Jiao Tong University, Shanghai, China.

**Keywords:** inflammatory bowel disease, SETD2, adipose-derived mesenchymal stem cell, Grem1, IL-10

## Abstract

**Rationale:** Inflammatory bowel disease (IBD) is a relapsing and idiopathic disorder. The low therapeutic efficacy of IBD urgently prompts us to seek new treatment methods.

**Methods and Results:** In this study, we report an adipose-derived mesenchymal stem cell (AT-MSC)-based treatment strategy in which AT-MSCs specifically deliver BMP inhibitor Grem1 and anti-inflammatory factor IL-10 to inflammatory colon tissues in SETD2 deficient dextran sulfate sodium (DSS)-induced colitis mouse models. Targeted delivery of Grem1 reduced colitis by promoting intestinal stem cell regeneration and enhancing mucosal regenerative capacity. Furthermore, targeted delivery of IL-10 reduced colitis by reducing inflammatory cytokines.

**Conclusion:** Our AT-MSCs based therapeutic strategy effectively mitigated IBD. This study has deepened our understanding of IBD therapy and provided a theoretical foundation for its clinical treatment.

## Introduction

Inflammatory Bowel Disease (IBD) is a complex disease characterized by chronic and heterogeneous manifestations, that can be induced by interacting genomic, environmental, microbial and immunological factors^1^, ^2^. IBD is an umbrella term that comprises ulcerative colitis, Crohn's disease, and IBD- unclassified^3^. IBD has increased in general and in childhood over the past 50 years. IBD is caused by the disruption in homeostasis between the intestinal epithelium, commensal bacteria, and mucosal immune system^3^. Nevertheless, the molecular mechanisms of IBD remain largely unclear.

The last few years have seen an expansion in IBD therapeutic options, with pharmacotherapy, biologics, and surgical resection being conventional treatments for IBD^4^. The first drugs used in IBD therapy were mesalamines and corticosteroids^5^, ^6^. However, many patients do not respond to those conventional treatments^7^ and IBD cannot be completely cured and is easy to relapse. It has been reported that IL-10 family cytokines play important roles in regulating the homeostatic states of the immune system and the intestinal epithelia^8^, ^9^. Besides, intestinal stromal cells produce BMP inhibitor Grem1 to promote Intestinal Stem Cells (ISCs) proliferation^10^ and Grem1 regulates epithelial cell fate in intestinal regeneration^11^. These results suggest that the using IL-10 and Grem1 may serve as a potential therapeutic strategy to overcome the problems of low therapeutic efficacy. Therefore, efficient immunotherapies specifically targeting IBD are urgently needed.

Mesenchymal stem cells (MSCs) regulate immune and tissue homeostasis, including IBDs^12^. MSCs can be isolated from bone marrow (BM-MSCs), adipose tissue(AT-MSC), umbilical cord blood( UCB-MSCs), human amniotic tissue (HA-MSCs), and gingiva tissues(GMSCs)^13^. AT-MSCs are more accessible, easier to collect, can be cultured for a longer period *in vitro*, and bear minimal risk to the donor and no ethical concerns^14^, ^15^. However, clinical trials have generated disappointing results, with only 30%-60% of IBD patients responding to treatment as of the latest clinical trials^16^. Notably, MSCs are emerging as promising cellular vehicles with immuno-modulatory and tissue-repair functions. With the advancement of MSC science, a variety of MSC-based drugs have been developed for IBD therapy^17^. However, the targeted delivery of IL-10 or Grem1 by MSCs for the treatment of IBD has not been applied^16^.

Besides, epigenetics plays an important role in the pathogenesis of IBD. Epigenetics is the study of mechanisms that influence transcription without modification to the genetic sequence, which includes DNA methylation, histone modification, and non-coding RNAs^18^, ^19^. SETD2 is the histone H3 lysine 36 (H3K36) methyltransferases mediating H3K36me3 modification and is important for many biological processes such as transcriptional regulation, DNA damage repair, and alternative splicing^20^-^22^. Our previous studies have reported that SETD2 modulates oxidative stress to attenuate experimental colitis. Thus, we sought to elucidate the therapeutic effect of targeting delivery of Grem1 and IL-10 in SETD2-deficient and DSS-induced inflammatory bowel disease.

In our study, we firstly elucidated that loss of Setd2 reduces ISCs proliferation and exacerbates DSS-induced colitis in mice. Next, we successfully delivered BMP inhibitor Grem1 and anti-inflammatory factor IL-10 to inflammatory colon tissues by AT-MSCs and reduced experimental colitis.

## Results

### Loss of Setd2 in ISCs exacerbates DSS-induced colitis in mice

**Figure 1:**
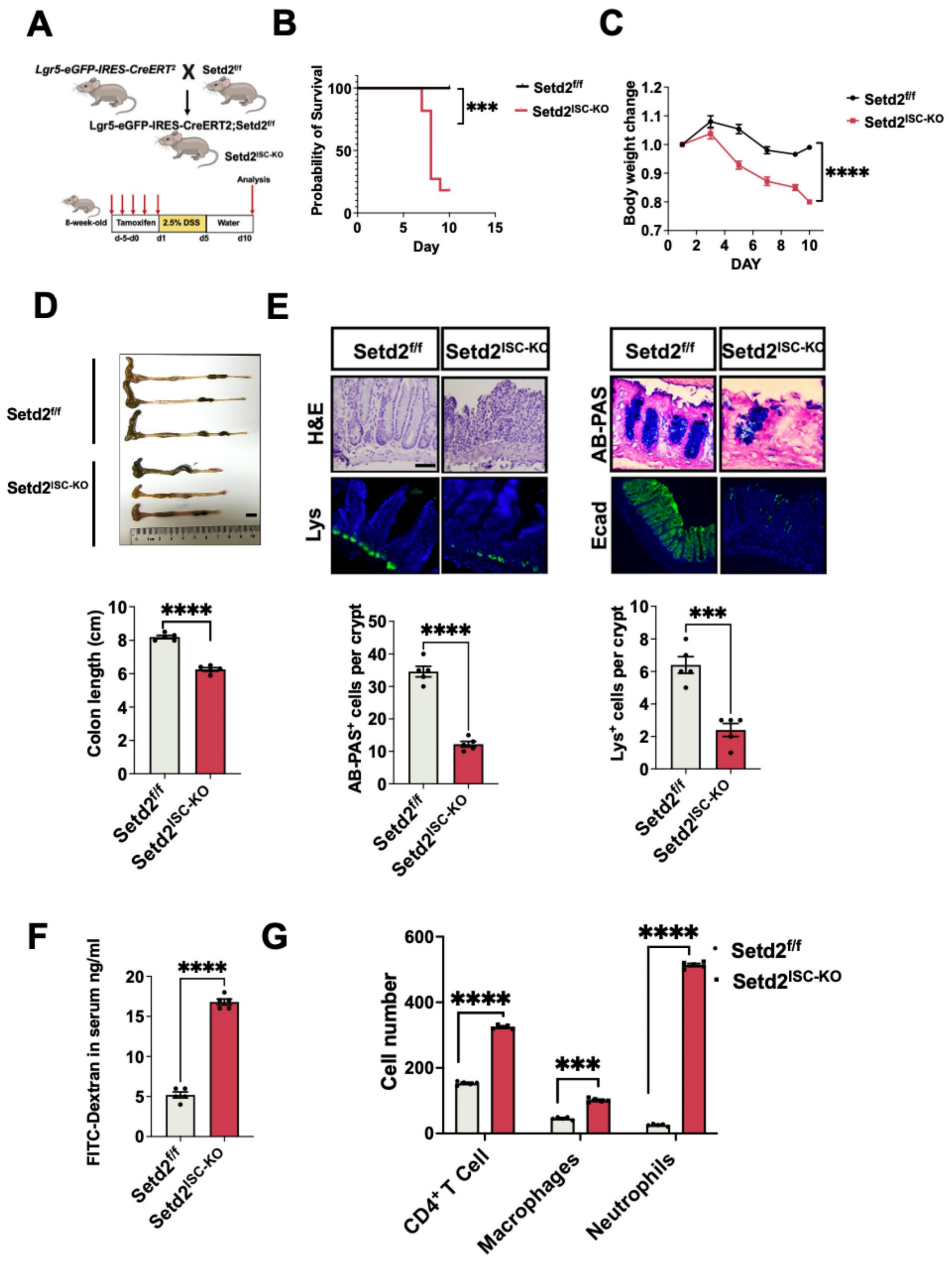
** Loss of Setd2 in ISCs exacerbates DSS-induced colitis in mice.** (A) Schematic representation of the DSS protocol used to induce acute colitis in Setd2^ISC-KO^ and Setd2^f/f^ mice. (B) Kaplan-Meier survival curves of mice used in this study. Comparisons of survival curves were made by the log-rank (Mantel-Cox) test. (C, D) Setd2^ISC-KO^ and Setd2^f/f^ mice were fed 2.5% DSS in drinking water, and loss of body weights (E) and colon length (F) are recorded (n =5 per genotype). Scale Bars: 1 cm. (E) H&E-stained sections of colon tissues collected on day 11 from 2.5% DSS-treated Setd2^ISC-KO^ and Setd2^f/f^ mice (n =5 per genotype) (Scale Bars: 100 μm). Lysozyme (Lys; Paneth cells) staining (Scale Bars: 50 μm) in the small intestine, Alcian Blue-Periodic acid Schiff (AB-PAS; goblet cells) staining in the colon (Scale Bars: 100 μm), E-cadherin staining (Scale Bars: 100 µm) in colon sections from DSS-treated (5 d) Setd2^ISC-KO^ and Setd2^f/f^ mice. Data represent means ± S.D., and statistical significance was determined by a Student t-test. *, p <0.05; **, p < 0.01; ***, p < 0.001. (F) Colonic permeability was assessed by measuring the concentration of FITC-dextran in the blood serum (n =5 per genotype). (G) Detection of the number of the immune cells in the colon of Setd2^ISC-KO^ and Setd2^f/f^ mice after DSS treatment by flow cytometry. (n = 5 per genotype).

First, we crossed Setd2-flox mice (Setd2^f/f^ mice) with Lgr5-eGFP-IRES-CreERT^2^ mice to obtain an intestinal stem cells-specific tamoxifen-inducible Setd2 knockout mouse strain (Lgr5-eGFP-IRES-CreERT2 mice, hereinafter referred to as Setd2^ISC-KO^ mice). As expected, Setd2 was efficiently ablated in the intestinal epithelium of Setd2^ISC-KO^ mice (Figure 1A, Figure S1A). We assessed the consequences of Setd2 loss in ISCs in colitis by challenging Setd2^f/f^ and Setd2^ISC-KO^ mice with 2.5% DSS after tamoxifen administration. After DSS administration, Setd2^ISC-KO^ mice had a lower survival rate and lost more body weight than Setd2^f/f^ mice (Figure 1B-1C), suggesting that Setd2 disruption enhanced inflammation and intestinal damage, as body weight loss is one of the characteristics of the severity of DSS-induced colitis^23^. Macroscopic dissection revealed significantly shorter colons in the Setd2^ISC-KO^ mice compared with the Setd2^f/f^ mice (Figure 1D). At 10 days post DSS treatment, the Setd2^f/f^ colon showed minimal to mild inflammation, while the colon from Setd2^ISC-KO^ mice displayed moderate to severe inflammation, with many areas of complete crypt loss and erosions. Besides, in contrast to controls, the Setd2^ISC-KO^ colons showed an excess inflammatory response with a loss of goblet cells and paneth cells, partially disrupted E-cadherin (Figure 1E). Furthermore, the DSS-treated Setd2^ISC-KO^ colons produced significantly more proinflammatory cytokines than the colons from the Setd2^f/f^ mice (Figure S1B). Intestinal permeability was markedly increased in Setd2^ISC-KO^ mice, evidenced by the enhanced fluorescence in the serum of DSS-treated Setd2^ISC-KO^ mice fed with FITC-labeled dextran (Figure 1F). Next, we analyzed immune cell infiltration by performing flow cytometry. As expected, Setd2^ISC-KO^ colons showed a higher number of CD4+T cells, neutrophils, and macrophages than controls (Figure 1G). Collectively, these results demonstrate that loss of Setd2 in ISCs aggravates DSS-induced colitis in mice.

### Loss of Setd2 reduces ISCs proliferation in mice

**Figure 2:**
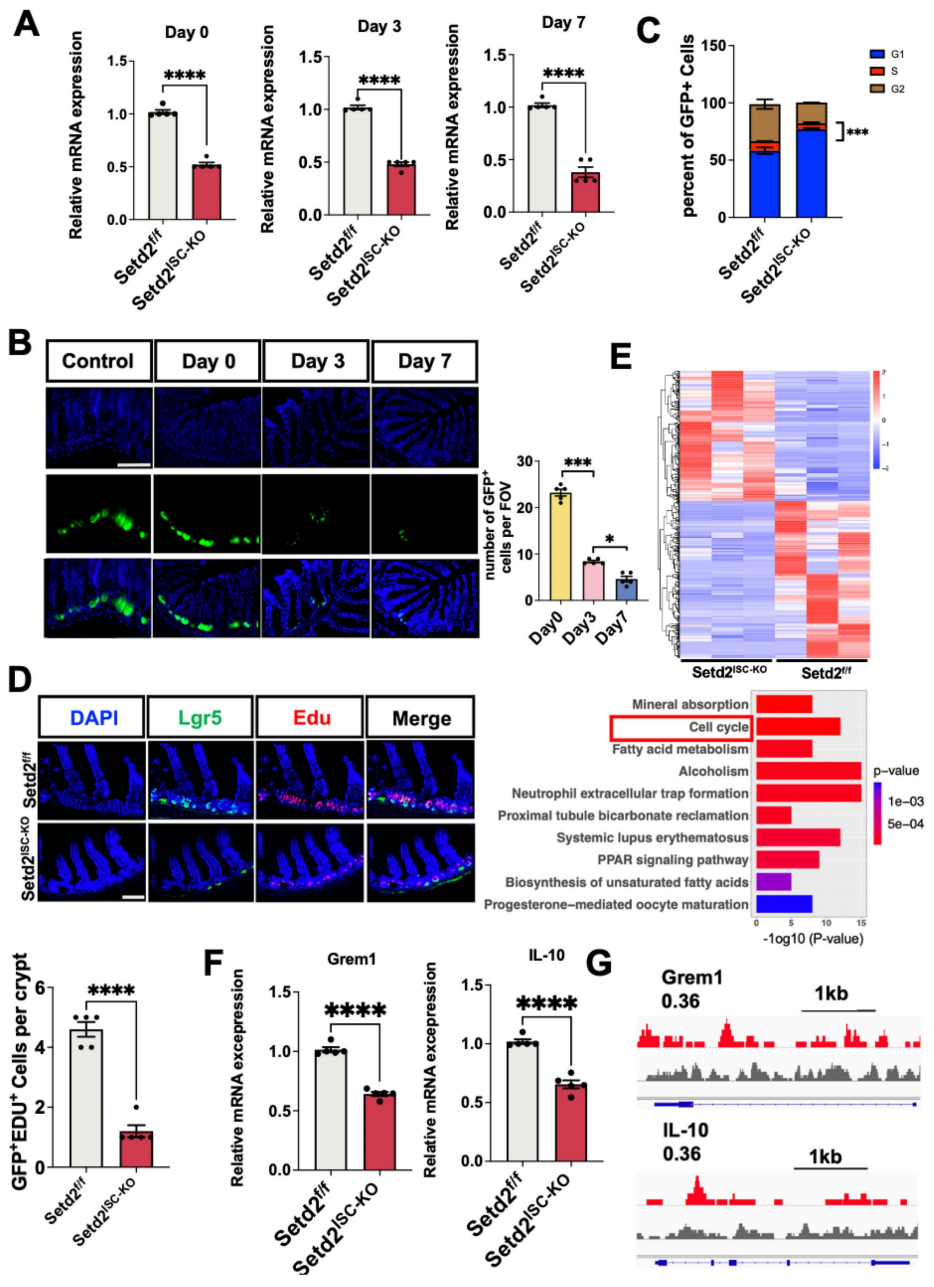
** Loss of Setd2 reduces ISCs proliferation in mice.** (A) Relative mRNA expression levels of Setd2 in the whole colon of Setd2^ISC-KO^ and Setd2^f/f^ mice were determined by RT-qPCR on day 0, day 3, and day 7 (n = 5 per genotype). (B) Numbers of GFP+ cells from a continuous 7 crypts of *Setd2*^ISC-KO^ mice after tamoxifen induction. *n* = 5 mice for each day. (Scale Bars: 100 μm). (C) Cell cycle analysis of Lgr5+ ISCs from Setd2^ISC-KO^ and Setd2^f/f^ mice on day 3. Data are presented as mean ± SE (n = 3). Student's t-test, *P < 0.05, **P < 0.01, ***P < 0.001. (D) EdU staining of intestine sections from Setd2^ISC-KO^ and Setd2^f/f^ mice on day 3 (n = 5 per genotype). (E) Gene Ontology (GO) analysis of downregulated genes in knockout (KO) small intestinal crypts on day 3, obtained from RNA-seq (n = 3). Among a total of 17236 genes detected, 324 genes were up-regulated, and 390 genes were down-regulated (Fold change >1.25) in Setd2^ISC-KO^ ISCs. Gene Ontology (GO) term analysis indicated that there was a significant enrichment of genes linked to cell cycle. (F) Relative mRNA expression levels of Grem1 and IL-10 in the whole colon of Setd2^ISC-KO^ and Setd2^f/f^ mice were determined by RT-qPCR (n = 5 per genotype). (G) Snapshot of H3K36me3 ChIP-Seq signals at the Grem1 and IL-10 in IECs isolated from Setd2^f/f^ mice.

Next, we examined whether Setd2 deficiency could affect ISCs proliferation. We assessed the consequence of Setd2 loss in ISCs at continuous time points after tamoxifen administration (Figure S2). Setd2 was efficiently ablated in the intestinal crypts of Setd2^ISC-KO^ mice (Figure 2A). Surprisingly, we observed the number of Lgr5+ cells in the Setd2^ISC-KO^ small intestine were reduced dramatically at day 3 (Figure 2B). Consistently, the cell cycle analysis^24^ of Lgr5+ ISCs showed that Setd2 knockout led to more ISCs arrested at the G1 phase (Figure 2C). In addition, fewer GFP+EdU+ cells^25^ were observed in Setd2^ISC-KO^ intestine, indicating that loss of Setd2 reduced the proliferation of Lgr5+ stem cells (Figure 2D). Furthermore, we also performed RNA sequencing (RNA-seq) analysis using RNA from GFP^high^Epcam^+^CD24^low^ ISCs from Setd2^ISC-KO^ mice and Setd2^f/f^ mice after tamoxifen injection without DSS administration^1^. Among a total of 17236 genes detected, 324 genes were up-regulated, and 390 genes were down-regulated (Fold change >1.25) in Setd2^ISC-KO^ ISCs. Gene Ontology (GO) term analysis indicated that there was a significant enrichment of genes linked to cell cycle (Figure 2E), which is consistent with previous observation (Figure 2D). Besides, we performed RT-qPCR, and the results showed that loss of Setd2 reduces the mRNA expression of Grem1 and IL-10 (Figure 2F). In addition, ChIP-Seq analysis revealed that direct H3K36me3 occupancies within Grem1 and IL-10 gene loci in Genome Browser tracks (Figure 2G). These results indicate that loss of Setd2 reduces the expression of Grem1, IL-10 and inhibits ISCs proliferation in mice.

### AT-MSCs selectively migrate to and reside in inflammatory colon

**Figure 3:**
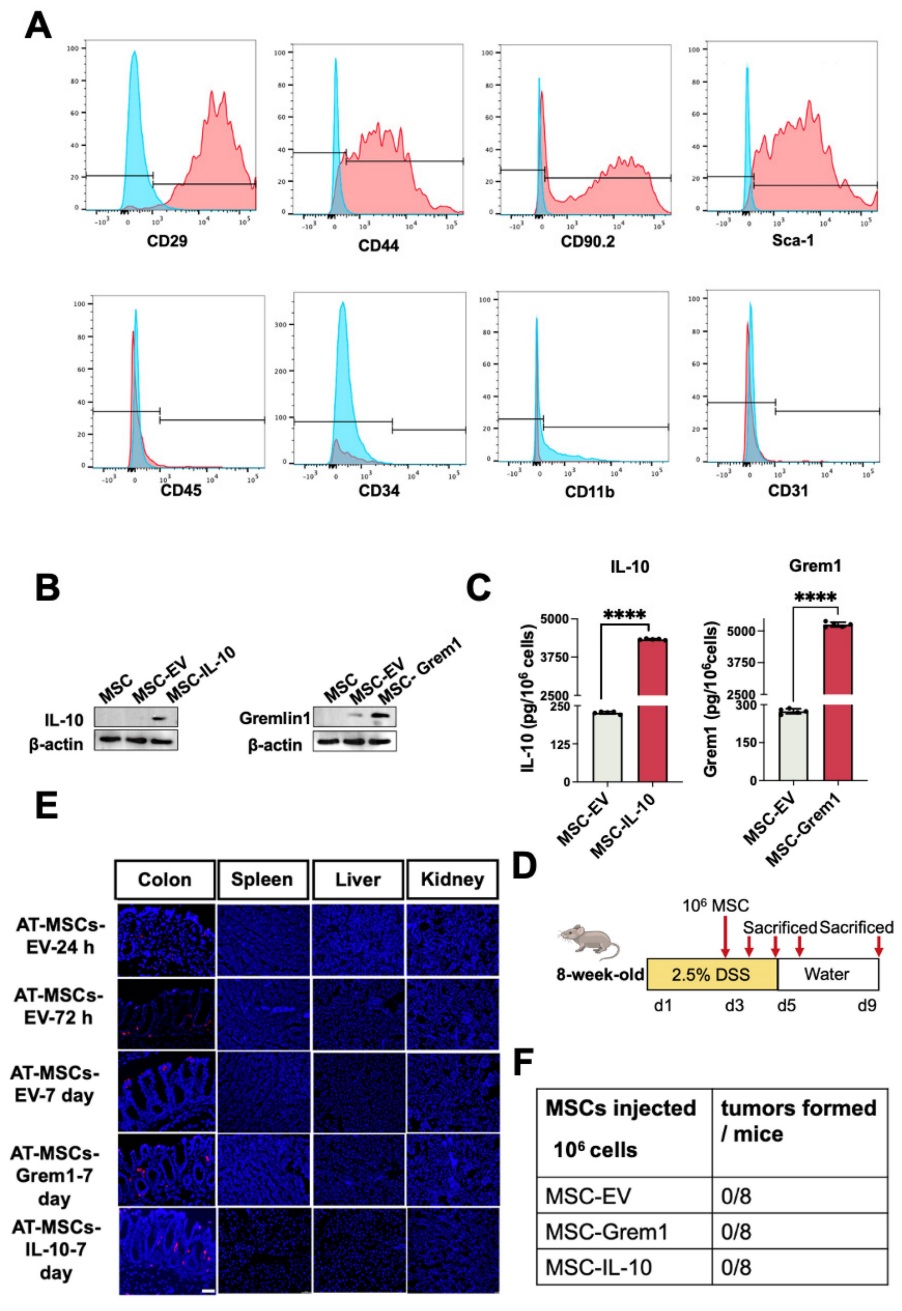
** AT-MSCs selectively migrate to and reside in inflammatory colon.** (A) Characterization of AT-MSCs isolated from C57BL/6J mice by FACS. (B) Western-Blot analyses of MSCs overexpressing mCherry, IL-10 or Grem1. (C) ELISA of MSCs overexpressing mCherry, IL-10 or Grem1. (D) Experimental layout of AT-MSC treatment in the DSS-treated mice. The colitis model was induced by 2.5% DSS in drinking water for 5 consecutive days. Mice received an intravenous injection of 1×10^6^ AT-MSCs on day 3 (n = 3). (E) Colon and other organs were collected after the AT-MSCs injection (1×10^6^ cells per mouse). AT-MSCs-mCherry were detected in tissue sections by immunofluorescent staining (mCherry, red) at 24-, 72-hours and 7 days post-injection. AT-MSCs-Grem1 and AT-MSCs-IL-10 were detected in tissue sections by immunofluorescent staining at 7 days post-injection. Nuclei were stained with DAPI (blue). Scale bars, 50 µm. (F) AT-MSCs isolated from C57BL/6J mice were lentivirally transduced and intravenously injected to C57BL/6J mice and tumor formation was evaluated.

To test whether AT-MSCs in our study can serve as an ideal vehicle for delivery of therapeutic molecules to inflammatory colon tissues, we first isolated and characterized mouse AT-MSCs by flow cytometry analysis^15^. These cells expressed MSCs-specific markers, including CD29, CD44, CD90, and Sca-1, and they lacked myeloid cell marker CD11b, endothelial cell marker CD31, hematopoietic or endothelial progenitor cell marker CD34, or leukocyte marker CD45(Figure 3A), suggesting a high purity of the AT-MSCs. As we mentioned before, previous reports suggest the therapeutic potential of IL-10 and Grem1. Therefore, we engineered mCherry labeled AT-MSCs overexpressing IL-10 or Grem1 by lentiviral transduction. As expected, AT-MSCs expressed IL-10 or Grem1 (Figure 3B). Secretion of Grem1 and IL-10 were further evaluated by enzyme-linked immunosorbent assay (ELISA) (Figure 3C). The amount of secreted Grem1 and IL-10 was always within the range of 4,000-6,000 pg/10^6^ cells after 48-h culture for all subsequent experiments as determined by ELISA. AT-MSCs of C57BL/6 J mice origin were transduced to stably express mCherry by lentiviruses, and an injection of 1×10^6^ mCherry-labeled AT-MSCs were given to mice after 2.5% DSS administration^26^ via the tail vein (Figure 3D). To track AT-MSCs after injection, cells were injected intravenously on day 3 of DSS administration. Mice were sacrificed after the injection at different days and cryosections of multiple organs were analyzed. Labeled cells were enriched in the colitis, but not in other organs, including the spleen, liver and kidney (Figure 3E). Lentivirally transduced AT-MSCs did not show any tumorigenic ability throughout our entire study (Figure 3F). These results reveal that our genetically engineered AT-MSCs can specifically migrate to and reside in inflammatory colon tissues without distributing in normal organs, suggesting their potential as a safe and efficient drug vehicle to target inflammatory colon tissues.

### Targeted delivery of Grem1 by AT-MSCs effectively reduces DSS-induced colitis

**Figure 4:**
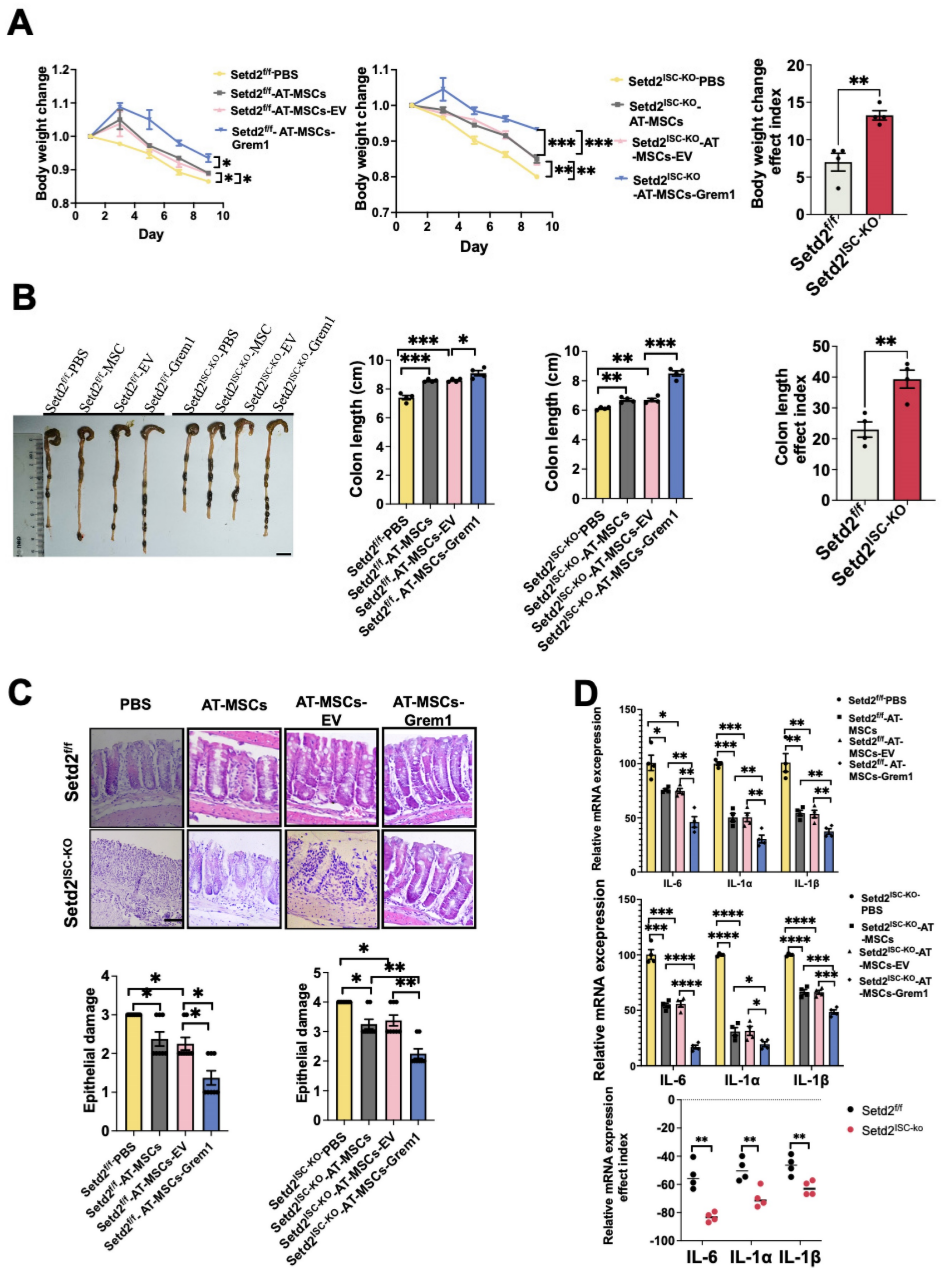
** Targeted delivery of Grem1 by AT-MSCs effectively reduces DSS-induced colitis.** (A) Setd2^ISC-KO^ and Setd2^f/f^ mice were fed 2.5% DSS in drinking water and injected 1×10^6^ MSCs and loss of body weight are recorded (n = 4) (Left). The efficacy indexes were assessed by Levene's test (right). (B) Setd2^ISC-KO^ and Setd2^f/f^ mice were fed 2.5% DSS in drinking water and injected 1×10^6^ MSCs and colon length are recorded (n = 4). The efficacy indexes were assessed by Levene's test. (C) H&E-stained sections of colon tissues collected on day 9 from Setd2^ISC-KO^ and Setd2^f/f^ mice were fed 2.5% DSS in drinking water and injected 1×10^6^ MSCs (Scale Bars: 100µm) and epithelial damage is recorded (n = 4). (D) Relative mRNA expression levels of pro-inflammatory cytokines in the whole colon of Setd2^ISC-KO^ and Setd2^f/f^ mice were fed 2.5% DSS in drinking water and injected 1×10^6^ MSCs (n = 4). The effect index of relative mRNA expression levels of pro-inflammatory cytokines in the whole colon of Setd2^ISC-KO^ and Setd2^f/f^ mice were fed 2.5% DSS in drinking water and injected 1×10^6^ MSCs. The efficacy indexes were assessed by Levene's test.

To examine the effects of MSC therapy on IBD, we first induced a colitis model and injected engineered AT-MSCs intravenously (Figure S3A)^26^. For Setd2^f/f^ mice, compared to the Setd2^f/f^-PBS group, the Setd2^f/f^-AT-MSCs group and Setd2^f/f^-AT-MSCs-EV group showed reduced body weight loss. And compared to the Setd2^f/f^-AT-MSCs group and Setd2^f/f^-AT-MSCs-EV group, the Setd2^f/f^-Grem1-AT-MSCs group showed reduced body weight loss. Moreover, for Setd2^ISC-KO^ mice, compared to the Setd2^ISC-KO^ -PBS group, the Setd2^ISC-KO^-AT-MSCs group and Setd2^ISC-KO^-AT-MSC-EV group showed reduced body weight loss. And compared to the Setd2^ISC-KO^-AT-MSCs group and Setd2^ISC-KO^-AT-MSC-EV group, the Setd2^ISC-KO^-Grem1-AT-MSCs group showed reduced body weight loss (Figure 4A). Surprisingly, the efficacy indexes^27^,^28^ of the Setd2^ISC-KO^ colitis mice after treatment were statistically significantly higher than Setd2^f/f^ mice (Figure 4A).

Besides, for Setd2^f/f^ mice, the average colon length was reduced to a lesser degree in the Setd2^f/f^-AT-MSCs group and Setd2^f/f^-AT-MSCs-EV group than Setd2^f/f^- PBS controls, and Setd2^f/f^ -Grem1-AT-MSCs group colon length was reduced to the least degree. Similarly, for Setd2^ISC-KO^ mice, the average colon length was reduced to a lesser degree in the Setd2^ISC-KO^-AT-MSCs group and Setd2^ISC-KO^-AT-MSC-EV group than in Setd2^f/f^- PBS controls, and Setd2^f/f^ -Grem1-AT-MSCs group colon length was reduced to the least degree (Figure 4B). And as expected, the efficacy indexes of the Setd2^ISC-KO^ colitis mice after treatment were statistically significantly higher than in Setd2^f/f^ mice (Figure 4B).

H&E sections indicated that epithelium loss and inflammatory cell infiltration were reduced in the Setd2^f/f^-AT-MSCs group and Setd2^f/f^-AT-MSCs-EV group than Setd2^f/f^- PBS controls, and more reduced in the Setd2^f/f^ -Grem1-AT-MSCs group than the Setd2^f/f^-AT-MSCs group and Setd2^f/f^-AT-MSCs-EV group. And for Setd2^ISC-KO^ mice, epithelium loss^29^ and inflammatory cell infiltration were reduced in the Setd2^ISC-KO^-AT-MSCs group and Setd2^ISC-KO^-AT-MSC-EV group than Setd2^ISC-KO^-PBS controls, and more reduced in the Setd2^ISC-KO^-Grem1-AT-MSCs group than Setd2^ISC-KO^-AT-MSCs group and Setd2^ISC-KO^-AT-MSC-EV group (Figure 4C).

Accordingly, Setd2^f/f^-AT-MSCs group and Setd2^f/f^-AT-MSCs-EV group colons produced prominently less proinflammatory cytokines and chemokines than the colons from the Setd2^f/f^-PBS. Setd2^f/f^-Grem1-AT-MSC colons produced prominently less proinflammatory cytokines and chemokines than the colons from Setd2^f/f^-AT-MSCs group and Setd2^f/f^-AT-MSCs-EV group. For Setd2^ISC-KO^ mice, Setd2^ISC-KO^-AT-MSCs group and Setd2^ISC-KO^-AT-MSC-EV group colons produced prominently less proinflammatory cytokines and chemokines than the colons from the Setd2^ISC-KO^-PBS; the Setd2^ISC-KO^+Grem1-AT-MSC group produced prominently fewer proinflammatory cytokines and chemokines than the colons from Setd2^ISC-KO^-AT-MSCs group and Setd2^ISC-KO^-AT-MSC-EV group (Figure 4D). For Grem1-AT-MSC groups, the efficacy indexes of the Setd2^ISC-KO^ colitis mice after treatment were statistically significantly higher than Setd2^f/f^ mice (Figure 4D). Thus, these results demonstrate that** t**argeted delivery of Grem1 by AT-MSCs effectively reduces colitis and shows higher efficacy in Setd2-deficient colitis.

### Targeted delivery of Grem1 by AT-MSC inhibits BMP signaling and increases ISCs number signaling

**Figure 5:**
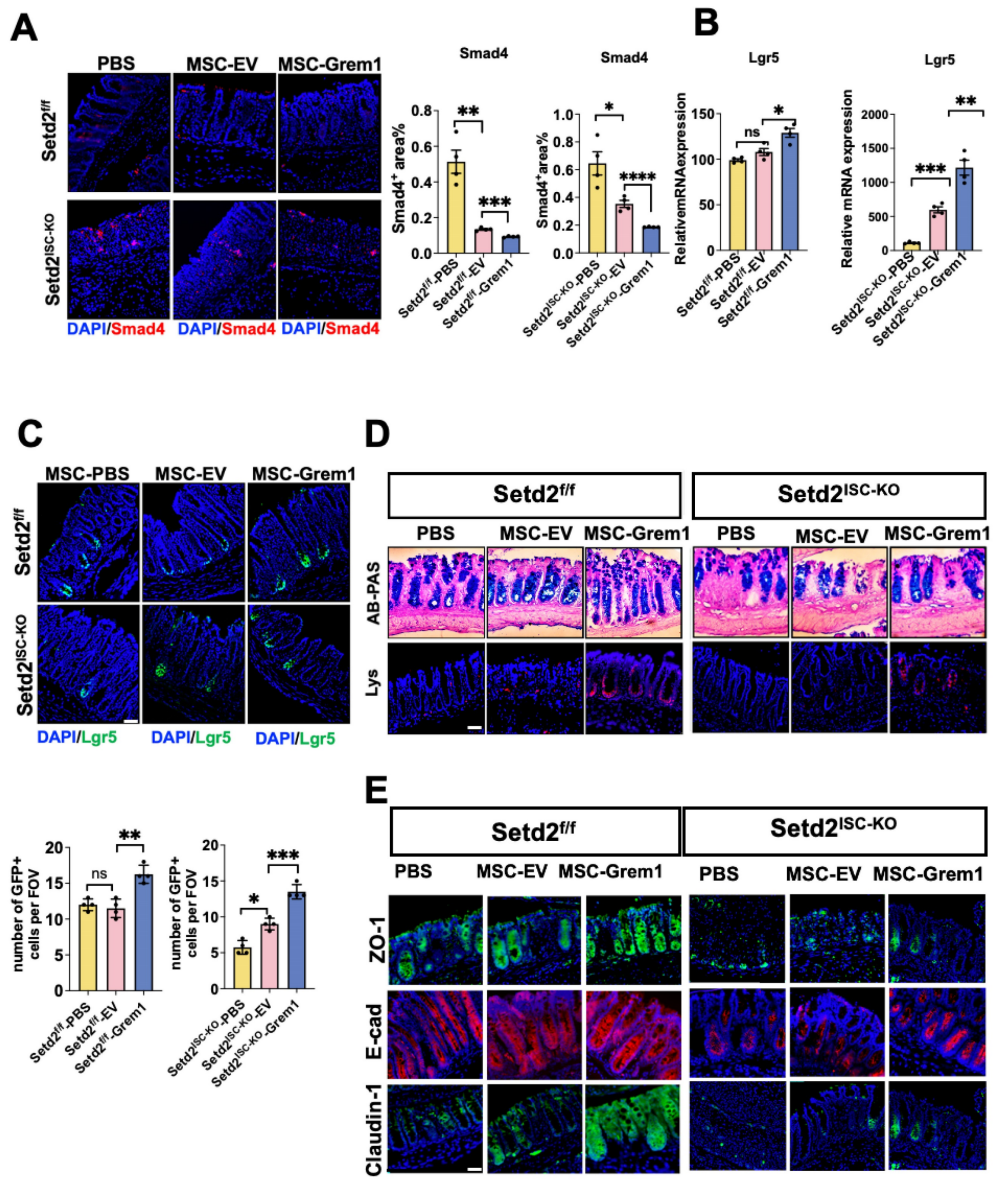
** Targeted delivery of Grem1 by AT-MSC inhibits BMP signaling and increases ISCs number signaling.** (A) Immunofluorescence staining of BMP4 of colon tissues collected on day 9 from Setd2^ISC-KO^ and Setd2^f/f^ mice were fed 2.5% DSS in drinking water and injected 1×10^6^ AT-MSCs. (n = 4 per genotype). Scale Bars: 100 μm. Data represent means ± S.D., and statistical significance was determined by a student t-test. *, p < 0.05; **, p < 0.01; ***, p < 0.001. (B) Relative mRNA expression levels of ISCs signature gene expression in the whole colon of Setd2^ISC-KO^ and Setd2^f/f^ mice were fed 2.5% DSS in drinking water and injected 1×10^6^ MSCs (n = 4 per genotype). Data represent means ± S.D. (C) Immunofluorescence staining of endogenous Lgr5-EGFP in the colon tissues collected on day 9 from Setd2^ISC-KO^ and Setd2^f/f^ mice were fed 2.5% DSS in drinking water and injected 1×10^6^ MSCs (n = 4 per genotype). Record of endogenous Lgr5-EGFP in the colon tissues collected on day 9 from Setd2^ISC-KO^ and Setd2^f/f^ mice were fed 2.5% DSS in drinking water and injected 1×10^6^ MSCs (n = 4 per genotype). (D) H&E-stained sections of colon tissues collected on day 9 from Setd2^ISC-KO^ and Setd2^f/f^ mice were fed 2.5% DSS in drinking water and injected 1×10^6^ MSCs. (n = 4 per genotype). Alcian Blue-Periodic acid Schiff (AB-PAS; goblet cells) staining in the colon, Lysozyme (Lys; Paneth cells) staining in the colon. Scale Bars: 100 μm. Data represent means ± S.D., and statistical significance was determined by a Student t-test. *, p < 0.05; **, p < 0.01; ***, p < 0.001. (E) Immunofluorescence staining of ZO-1, E-cad and Claudin-1 of colon tissues collected on day 9 from Setd2^ISC-KO^ and Setd2^f/f^ mice were fed 2.5% DSS in drinking water and injected 1×10^6^ MSCs. (n = 4per genotype). Scale Bars: 100 μm. Data represent means ± S.D., and statistical significance was determined by a Student t-test. *, p < 0.05; **, p < 0.01; ***, p < 0.001.

It has been reported that Grem1 blocks BMP function^30^, and BMP restricts stemness of intestinal Lgr5+ stem cells^31^. To study the mechanism of targeted delivery of BMP inhibitor Grem1 by AT-MSC, we tested BMP signaling. Reduced smad4 immunofluorescence and elevated stem cell signature gene mRNA confirmed inhibited BMP signaling in Grem1 targeted delivered colitis (Figure 5A, 5B, S4A). And targeted delivery of Grem1 by AT-MSCs increased Lgr5+ cells (Figure 5C), goblet cells and paneth cells number (Figure 5D, S4B). Besides, targeted delivery of Grem1 by AT-MSCs expedited epithelial Zo-1, E-cad Claodin-1 expression (Figure 5E) and decreased cell cycle arrest genes (Figure S4C). Together, these findings implicate that targeted delivery of Grem1 by AT-MSCs inhibits BMP signaling and increases ISCs number signaling.

### Targeted delivery of IL-10 by AT-MSC reduces DSS-induced colitis

**Figure 6:**
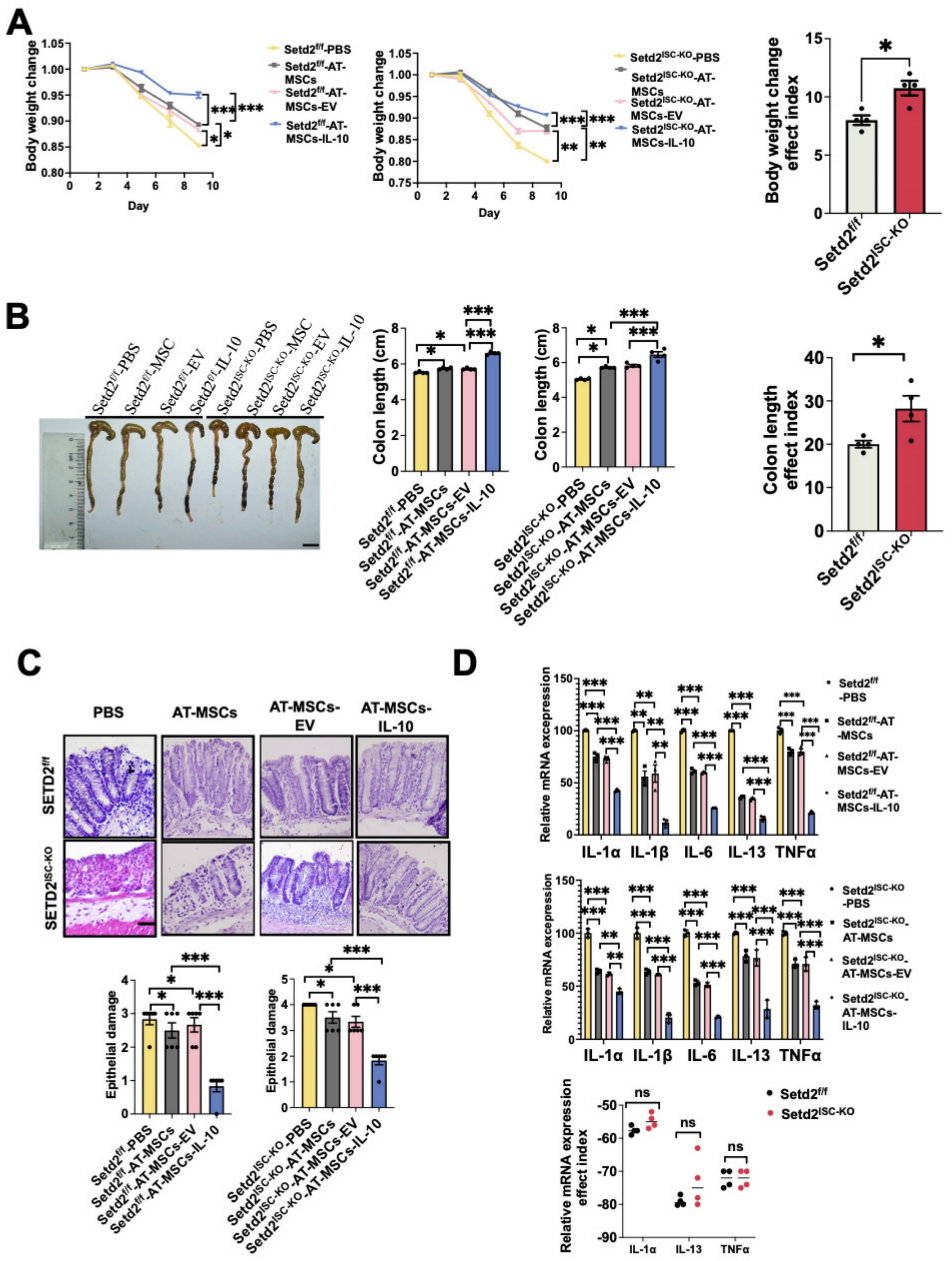
** Targeted delivery of IL-10 by AT-MSC reduces DSS-induced colitis.** (A) Setd2^ISC-KO^ and Setd2^f/f^ mice were fed 2.5% DSS in drinking water and injected 1×10^6^ MSCs and loss of body weight are recorded (n = 4) (Left). The efficacy indexes were assessed by Levene's test (Right). (B) Setd2^ISC-KO^ and Setd2^f/f^ mice were fed 2.5% DSS in drinking water and injected 1×10^6^ MSCs and colon length are recorded (n = 4). The efficacy indexes were assessed by Levene^'^s test (Right). (C) H&E-stained sections of colon tissues collected on day 9 from Setd2^ISC-KO^ and Setd2^f/f^ mice were fed 2.5% DSS in drinking water and injected 1×10^6^ MSCs (Scale Bars: 100 μm). (D) Relative mRNA expression levels of pro-inflammatory cytokines in the whole colon of Setd2^ISC-KO^ and Setd2^f/f^ mice were fed 2.5% DSS in drinking water and injected 1×10^6^ MSCs (n = 4). The effect index of relative mRNA expression levels of inflammatory mediators in the whole colon of Setd2^ISC-KO^ and Setd2^f/f^ mice were fed 2.5% DSS in drinking water and injected 1×10^6^ MSCs. The efficacy indexes were assessed by Levene's test.

To expand treatment options, we elucidated the therapeutic effect of targeting delivery of IL-10. First, the colitis model was induced, and IL-10 engineered MSCs were injected intravenously (Figure S5A). For both Setd2^f/f^ mice and Setd2^ISC-KO^ mice, compared to the PBS group, AT-MSC group and AT-MSC-EV group showed reduced body weight loss. And compared to the AT-MSCs group and AT-MSCs-EV group, the AT-MSCs-IL-10 group showed reduced body weight loss. And efficacy indexes of body weight loss of the Setd2^ISC-KO^ colitis mice after treatment were not statistically different from the efficacy indexes of Setd2^f/f^ mice (Figure 6A).

Moreover, for Setd2^f/f^ mice, the average colon length was reduced to a lesser degree in the Setd2^f/f^-AT-MSCs group and Setd2^f/f^-AT-MSCs-EV group than Setd2^f/f^- PBS controls, and Setd2^f/f^-AT-MSCs-IL-10 group colon length was reduced to the least degree. Similarly, for Setd2^ISC-KO^ mice, the average colon length was reduced to a lesser degree in the Setd2^ISC-KO^-AT-MSCs group and Setd2^ISC-KO^-AT-MSCs-EV group, and Setd2^ISC-KO^-AT-MSCs-IL-10 group colon length was reduced to the least degree (Figure 6B). The efficacy indexes of colon length of the Setd2^ISC-KO^ colitis mice after treatment were not statistically different from the efficacy indexes of Setd2^f/f^ mice.

And H&E sections indicated that epithelium loss and inflammatory cell infiltration were reduced in the Setd2^f/f^-AT-MSCs group and Setd2^f/f^-AT-MSCs-EV than Setd2^f/f^- PBS controls, and more reduced in the Setd2^f/f^-AT-MSCs-IL-10 group. And for Setd2^ISC-KO^ mice, epithelium loss and inflammatory cell infiltration were reduced in the Setd2^ISC-KO^-AT-MSCs group and Setd2^ISC-KO^-AT-MSCs-EV group than Setd2^ISC-KO^-PBS controls, and more reduced in the Setd2^ISC-KO^-AT-MSCs-IL-10 group (Figure 6C).

Accordingly, Setd2^f/f^-AT-MSCs group and Setd2^f/f^-AT-MSCs-EV group colons produced prominently less proinflammatory cytokines and chemokines than the colons from the Setd2^f/f^-PBS. Setd2^f/f^-AT-MSCs-IL-10 group colons produced prominently fewer proinflammatory cytokines and chemokines than the colons from Setd2^f/f^-AT-MSCs group and Setd2^f/f^-AT-MSCs-EV. For Setd2^ISC-KO^ mice, Setd2^ISC-KO^-AT-MSCs group and Setd2^ISC-KO^-AT-MSCs-EV group colons produced prominently fewer proinflammatory cytokines and chemokines than the colons from the Setd2^ISC-KO^-PBS; the Setd2^ISC-KO^-AT-MSCs-IL-10 group colons produced prominently less proinflammatory cytokines and chemokines than the colons from Setd2^ISC-KO^-AT-MSCs group and Setd2^ISC-KO^-AT-MSCs-EV group colons (Figure 6D). And the efficacy indexes of chemokines of the Setd2^ISC-KO^ colitis mice after treatment were not statistically different from the efficacy indexes of Setd2^f/f^ mice (Figure 6D). These results reveal that targeted delivery of IL-10 by AT-MSCs reduces DSS-induced colitis.

## Discussion

In our study, we demonstrated that targeted delivery of Grem 1 by AT-MSCs inhibits the BMP signaling pathway in intestinal epithelial cells, promotes the proliferation of intestinal stem cells, facilitates intestinal epithelial repair, suppresses inflammation, and enhances the therapeutic efficacy for colitis. Particularly in SETD2-deficient colitis, the therapeutic effect of targeting deliver Grem1 is significantly higher than that of DSS-induced colitis. Besides, in our study, we did not observe the formation of intestinal polyps or the induction of colon cancer because of targeted delivery of Grem1. Furthermore, we also demonstrated that targeted delivery of IL-10 by AT-MSC reduces colitis without forming intestinal polyps or inducing colon cancer. This suggests that targeting delivery of Grem1 or IL-10 for IBD patients may improve treatment efficacy.

Over the past 20 years, achieving complete mucosal repair has gradually been recognized as a key therapeutic objective in the treatment of IBD^32^-^34^. It has been reported that Grem1 regulates epithelial cell fate in intestinal regeneration^11^. Based on this, we elucidate that targeted delivery of Grem1 by AT-MSCs could effectively mitigate IBD. Furthermore, the treatment of IBD has relied almost exclusively on immunosuppression^35^. In this regard, we also explored that AT-MSCs targeted delivery of IL-10 can significantly alleviate colitis in DSS-induced colitis mouse models. IL-10 is an effective anti-inflammatory cytokine, however, IL-10 has a short serum half-life (1.1-2.6 h) with chemical and physical instability in circulation, which inevitably activates leukocytes and leads to patient harm and reduced therapeutic efficacy following systemic IL-10 injections^36^, ^37^. Our AT-MSCs based therapeutic strategy has partly overcome short half-life drawbacks of traditional treatments for IBD. Targeted delivery of IL-10 by AT-MSCs can maintain its presence *in vivo* for up to 7 days. Besides, we also explored that loss of Setd2 in ISCs exacerbates DSS-induced colitis in mice and **t**argeted delivery of Grem1 by AT-MSCs shows higher efficacy in Setd2-deficient colitis. Thus, targeting delivery of Grem1 for Setd2-deficient IBD patients is a strategy that could be used in future clinical applications.

The major safety issue related to MSCs-based therapy is the risk of cell transformation and tumor formation. Here, we did not observe the formation of intestinal polyps or the induction of intestinal cancer because of targeted delivery of Grem1 or IL-10 (Figure 3E)^38^. Taken together, our study provides novel insight into the therapeutic approach of IBD by achieving mucosal repair and immunosuppression, offering a solution to the urgent unmet need for IBD patients.

## Materials and Methods

### Animal experiments

All mice were maintained in a specific-pathogen-free (SPF) facility, and mouse experimental protocols were approved by the Renji Hospital Animal Care and Use Committee (202201027). Lgr5-EGFP-IRES-CreERT2 mice were generated as previously reported^1^and gifted by Prof. Jun Qin and Prof. Huawei Li. Setd2-flox mice were purchased from Shanghai Biomodel Organism Co. These strains were interbred to generate the experimental cohorts, which include the following genotypes: For induction of Lgr5-eGFP-IRES-creERT2-mediated recombination, mice were received 1 dose of tamoxifen 100 mg/kg by gavage for 5 consecutive days. Lgr5-eGFP-IRES-CreERT2; Setd2^f/f^ mice were harvested at the indicated time for intestinal histology investigation. Mice that lose more than 20% of their weight within one week will be euthanized and recorded as deceased. All the mice were maintained on a C57BL/6J background, and littermates with the same treatment were used for control experiments. For permeability experiments, 2-month-old mice were fasted for 4 hours and then treated with FITC-conjugated dextran (500 mg kg-1 body weight). The fluorescence intensity was determined using a FITC-dextran standard curve. To induce colitis, mice at 2 months of age were fed 2.5% DSS (molecular weight, 36-50 kDa; MP Biomedicals) for 5 days, followed by regular drinking water. Body weight was recorded daily. The AT-MSC group received a single injection of 1 × 10^6^ cells in 250 μL of phosphate-buffered saline (PBS) on day 3 via the tail vein, while the DSS group received the same volume of PBS. Score of each mouse were recorded daily according to standard protocols. Mice were sacrificed on day 9, and samples were collected.

### Antibodies

For histological analysis, Lys (ab36362, abcam), Claudin-1 (2H10d10, Invitrogen), ZO-1 (SA243690, Invitrogen), E-cadherin (#3195, CST). For Flow Cytometry, CD24 (25-0242-82, Thermo Fisher Scientific), EpCAM (17-5791-82, eBioscience), 7AAD (A1310, Thermo Fisher Scientific), Sca-1 (D7, eBioscience), CD29 (102213, Biolegend), CD45 (104, eBioscience), CD90.2 (553005, BDBioscience), CD11b (M1/70, Biolegend), CD31(390, Biolegend), CD34(560233, BDBioscience).

### DNA, RNA extraction

For DNA and RNA extraction, DNA was extracted with the QIAamp DNA Micro Kit (QIAGEN). The extracted DNA served as a template for amplifying both nuclear and mitochondrial genes. RNA was extracted by using the RNeasy Kit (QIAGEN), which included on-column DNase treatment (QIAGEN). cDNA synthesis was conducted using the iScript cDNA Synthesis Kit (Bio-Rad).

### Protein lysates and western blotting

Total proteins were extracted by directly lysing organoids in Laemmli sample buffer. The proteins were separated using SDS-PAGE and subsequently transferred to Immobilon Polyscreen PVDF transfer membranes (#IPVH00010, Merck Millipore) or Amersham Protan nitrocellulose membranes (#10600001 GE Healthcare Life Sciences). Western blot analysis was performed with primary antibodies recognizing for detection.

### Histological analyses

For frozen sections, the intestine was isolated from the indicated mice, washed with cold PBS, and fixed in 4% formaldehyde for 1 hour at room temperature. The tissues were then immersed in 20% sucrose overnight at 4 °C, followed by embedding in OCT (Optimal Cutting Temperature) compound (Sakura), freezing at -80 °C, and sectioning. For paraffin sections, the isolated intestine was fixed in 4% formaldehyde overnight at 4 °C, then embedded in paraffin and sectioned.

For immunofluorescence, frozen sections were immersed in phosphate-buffered saline (PBS) to remove the optimal cutting temperature (OCT) compound, followed by antigen retrieval using citrate buffer in a microwave oven for 15 minutes. The sections were then permeabilized with 0.1% Triton X-100 at 4 °C for 15 minutes and subsequently blocked with a solution of 3% bovine serum albumin (BSA) and 0.01% Triton X-100 at room temperature for 1 hour. The sections were incubated with the primary antibody at 4 °C overnight. Fluorescein-labeled secondary antibodies (Life Technologies, 1:300) along with DAPI were applied for 1 hour at room temperature. Images were captured using an Olympus FV3000 Laser Scanning Microscope. For hematoxylin and eosin (H&E) staining, paraffin sections were deparaffinized in xylene and graded alcohols, then stained with hematoxylin and eosin according to the manufacturer's instructions. Images were obtained using a slide scanning system (KF-PRO-120, Jiang Feng Technology).

### Cell cycle and cell ratio analyses

For cell cycle analysis, crypts isolated from the indicated mice were dissociated in TrypLE (Invitrogen) for 30 minutes at 37°C. The single-cell suspension was washed twice with pre-cooled PBS and then fixed in pre-cooled 70% ethyl alcohol overnight at 4°C. After washing with pre-cooled PBS twice, the cells were gently resuspended in propidium iodide staining solution (Beyotime) and incubated at 37°C for 30 minutes. The stained DNA content of EGFP+ (Lgr5+) intestinal stem cells (ISCs) was examined using flow cytometry (CytoFlex LX, Beckman Coulter) and analyzed with Kaluza Analysis software. For cell ratio analysis, organoids were dissociated in TrypLE (Invitrogen) for 20 minutes at 37°C. The single-cell suspension was passed through a 40 μm cell strainer (BD Biosciences) and centrifuged for 3 minutes at 500g. The cells were gently resuspended in propidium iodide staining solution (Beyotime) and analyzed by flow cytometry (CytoFlex LX, Beckman Coulter) to determine the ratio of EGFP+ (Lgr5+) ISCs.

### RNA-seq for repeats and analysis

ISC-related RNA-seq was performed in-house. Total RNA was extracted from GFP^high^CD24^low^ ISCs from Lgr5-eGFP-IRES-creERT2; Setd2^f/f^ mice (corn-oil-treated, n = 3; tamoxifen-treated, n = 3) 5 d after the first gavage. Cells were isolated into a PCR tube containing lysis buffer. Reverse transcription was performed using SMART Scribe Reverse Transcriptase (Takara) in the presence of oligo-dT30VN, template-switching oligonucleotides, and betaine. The cDNA was amplified using Seq Amp DNA Polymerase (Takara), PCR primers, and 18 cycles of amplification. Following purification with Agencourt Ampure XP beads (Beckman Coulter), the product's size distribution and quantity were assessed on a Bioanalyzer using a High Sensitivity DNA Kit (Agilent Technologies). A total of 50 ng of the amplified cDNA was fragmented using the Covaris® M220 (Covaris). The cDNA fragments were end-repaired, A-tailed, and custom KAPA dual-indexed adapters were ligated using the KAPA HyperPrep Kit (Roche). The products were purified twice with Agencourt Ampure XP beads and amplified for 15 cycles, after which they were quantified again using a Bioanalyzer High Sensitivity DNA Kit. The library was then subjected to Illumina sequencing with paired-end 2x150 as the sequencing mode.

### EdU assay

To assess the proliferation of intestinal epithelial cells, 5-ethynyl-2-deoxyuridine (EdU, MedChemExpress) was administered via intraperitoneal injection at a dosage of 100 mg/kg to mice, 2 h prior to euthanasia. The intestines were subsequently collected, and EdU-positive cells were identified using an EdU staining kit (RiboBio).

### Quantification and statistical analysis

The data are presented as mean ± SEM. Statistical analyses were conducted using GraphPad Prism 5.0 (GraphPad Software, La Jolla, CA, USA). An unpaired two-tailed Student's t-test was employed for comparisons between two groups, while ANOVA was utilized for comparisons involving more than two groups, followed by the Tukey post hoc test. Statistical significance was defined as P < 0.05. Effect index = (total score before treatment-total score after treatment) /pre-treatment total score × 100%.

### Flow cytometry

Crypts from Lgr5-eGFP-IRES-CreERT2; Setd2^f/f^ mice were isolated. GFP^high^ CD24^low^ intestinal stem cells (ISCs) were sorted using fluorescence-activated cell sorting (FACS) on a MoFlo Astrios EQS (Beckman Coulter). To characterize adipose tissue-derived mesenchymal stem cells (AT-MSCs), adherent cells at the third to fourth passages were detached using 10 mM EDTA, washed with phosphate-buffered saline (PBS), and stained with antibodies for FACS analysis.

### FITC-dextran intestinal permeability assay

Intestinal permeability was assessed using a permeability probe, FITC-dextran 4KD (Sigma-Aldrich). Mice were fasted overnight and then orally administered FITC-dextran at a dosage of 600 mg/kg. After 4 hours, the fluorescence intensity of serum FITC-dextran was measured at an excitation wavelength of 480 nm and an emission wavelength of 520 nm using a SpectraMax M5 microplate reader (Molecular Devices).

### MSC culture and preparation

Adipose tissue-derived mesenchymal stem cells (AT-MSCs) were isolated from mouse subcutaneous adipose tissue using collagenase type I (Thermo Fisher Scientific) digestion and a plastic adherence technique, as previously described. Cells were then plated in alpha-minimum essential medium (α-MEM) supplemented with 10% fetal bovine serum (FBS) and 1% penicillin/streptomycin.

### *In vivo* tracking of AT-MSCs

For mCherry labeling of AT-MSCs, the cells were transfected with a mCherry-labeled lentiviral vector and subsequently observed under a microscope. Mice were administered 2.5% DSS, and the labeled AT-MSCs were given intravenously. The mice were then sacrificed at specific time points.

### ELISA

Cell-free supernatants from lentivirus-transduced mouse adipose tissue-derived mesenchymal stem cells (AT-MSCs) were collected and stored in a refrigerator at -80°C until measurement.

### Lentivirus production and transduction in MSCs

cDNAs were cloned into lentiviral vectors. Lentiviruses were produced and titrated by OBiO Technology (Shanghai). Mesenchymal stem cells (MSCs) were infected with lentiviruses at a multiplicity of infection (MOI) of 60 in the presence of 8 mg/mL polybrene (Sigma-Aldrich). All lentiviral vectors contained green fluorescent protein (GFP), and a transduction efficiency of over 90% in MSCs was confirmed by GFP expression observed under microscopy prior to use. The successful expression of CXCL9 and OX40L in MSCs was routinely validated using enzyme-linked immunosorbent assay (ELISA) and fluorescence-activated cell sorting (FACS), respectively.

## Supplementary Material

Supplementary figures.
