# Supplementary material for: Targeted delivery of Grem1 and IL-10 separately by mesenchymal stem cells effectively mitigates SETD2-deficient inflammatory bowel disease
Source: Theranostics. 2025 Jan 13;15(6):2215–28. doi: 10.7150/thno.105876 (PMC11840741; doi:10.7150/thno.105876)
Supplement: Supplementary file 1 — Supplementary figures. [file thnov15p2215s1.pdf]

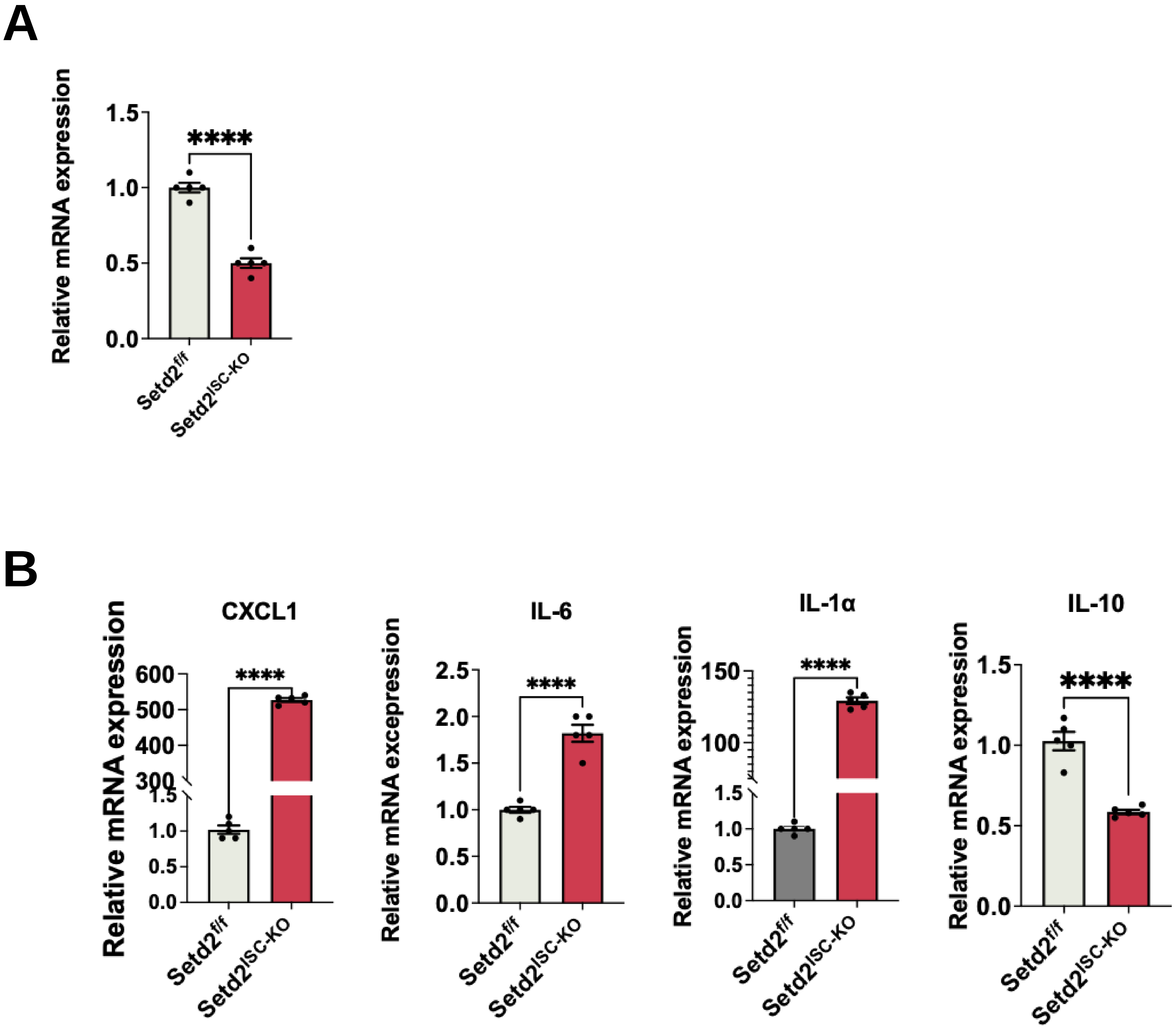

Figure S1. Loss of Setd2 in ISCs exacerbates DSS-induced colitis in mice

(A) Relative mRNA expression levels of Setd2 in whole colon of Setd2$^{ISC\text{-}KO}$ and Setd2$^{f/f}$ mice were determined by RT-qPCR (n =5 per genotype).

(B) Relative mRNA expression levels of pro-inflammatory cytokines in whole colon of Setd2$^{ISC\text{-}KO}$ and Setd2$^{f/f}$ mice were determined by RT-qPCR (n =5 per genotype).

A

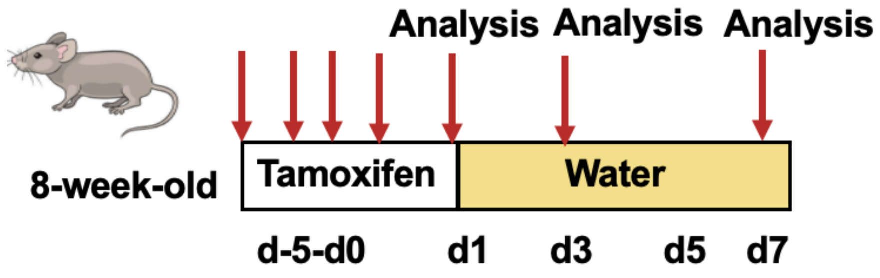

Figure S2. Loss of Setd2 reduces ISCs proliferation in mice.

(A) Experimental layout of Setd2$^{ISC\text{-}KO}$ mouse model. The model was induced by tamoxifen intraperitoneal injection for 5 consecutive days. (n=5).

A

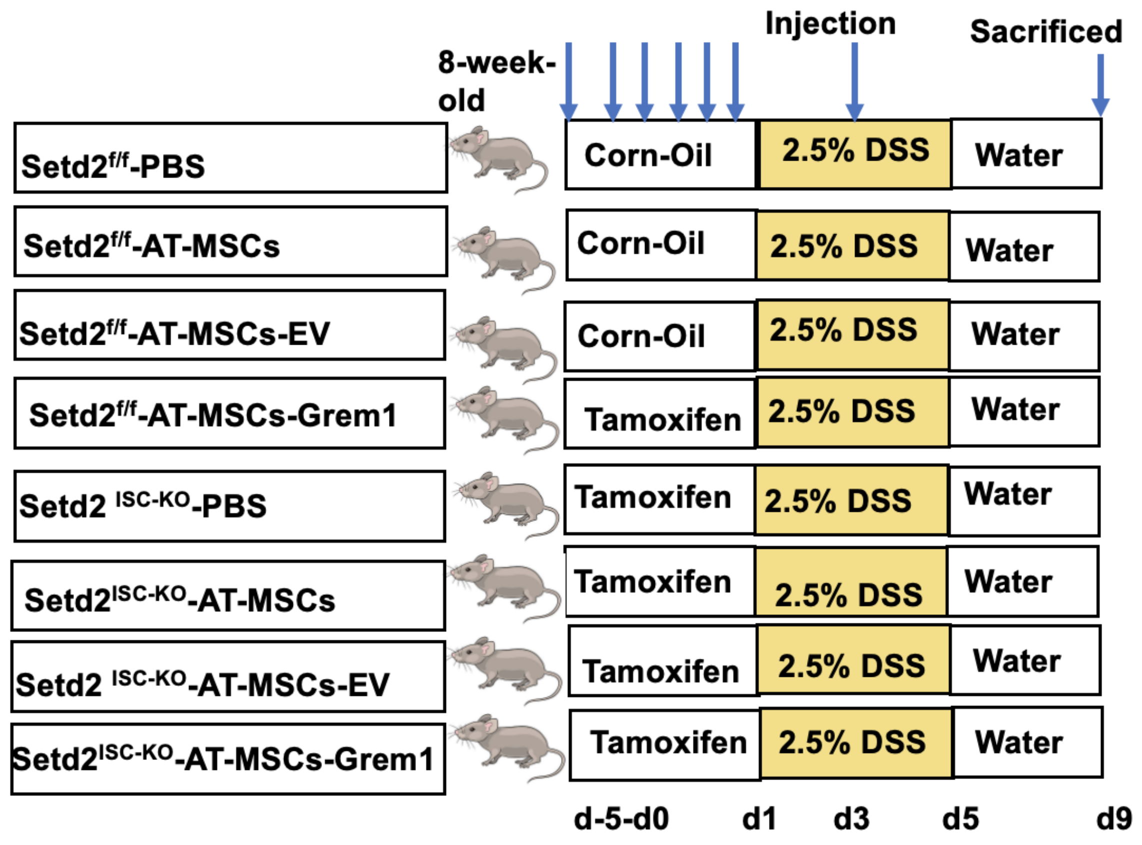

Figure S3. Targeted delivery of Grem1 by AT-MSC effectively reduces colitis

(A) Experimental layout of MSC-Grem1 treatment in the colitis. The colitis model was induced by one dose of tamoxifen and 2.5% DSS in drinking water for 5 consecutive days. Mice received an intravenous injection of 1×10^6 AT-MSCs (n=4) or PBS (n=4) on day 3.

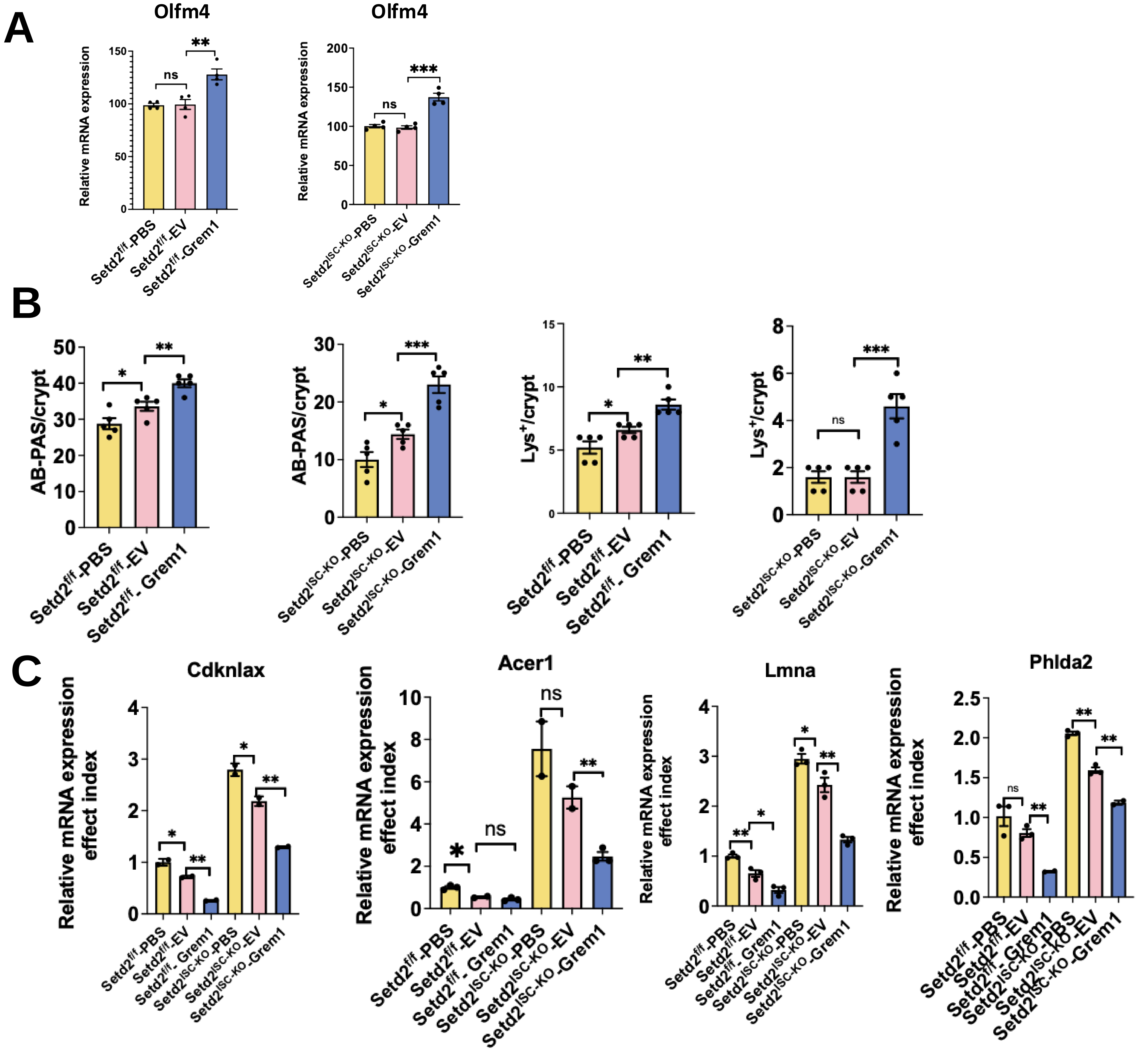

Figure S4. Targeted delivery of Grem1 by AT-MSC increases ISCs number and inhibits BMP signaling

(A) Relative mRNA expression levels of ISCs signature gene expression in the whole colon of Setd2$^{ISC\text{-}KO}$ and Setd2$^{f/f}$ mice were fed 2.5% DSS in drinking water and injected 1*10^6 MSCs (n=4 per genotype). Data represent means ± S.D.

(B) Record of colon tissues collected on day 9 from Setd2$^{ISC\text{-}KO}$ and Setd2$^{f/f}$ mice were fed 2.5% DSS in drinking water and injected 1*10^6 MSCs. (n = 4 per genotype) .

(C) Relative mRNA expression levels of cell cycle arrest genes in the Lgr5+ cells of colon from Setd2$^{ISC\text{-}KO}$ and Setd2$^{f/f}$ mice were fed 2.5% DSS in drinking water and injected 1*10^6 MSCs. Data represent means ± S.D., and statistical significance was determined by a Student t-test. *, p < .05; **, p < .01; ***, p < .001.

A

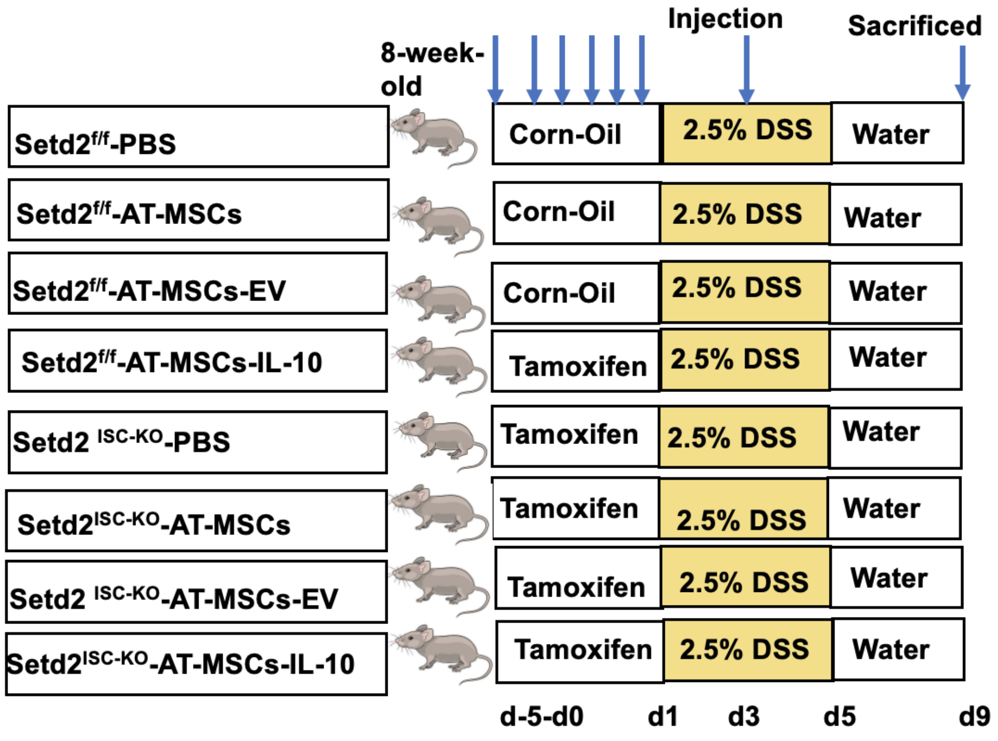

Figure S5. Targeted delivery of IL-10 by AT-MSC reduces colitis

(A) Experimental layout of MSC-IL-10 treatment in the colitis. The colitis model was induced by one dose of tamoxifen and 2.5% DSS in drinking water for 5 consecutive days. Mice received an intravenous injection of 1×10^6 AT-MSCs (n=4) or PBS (n=4) on day 3.

A

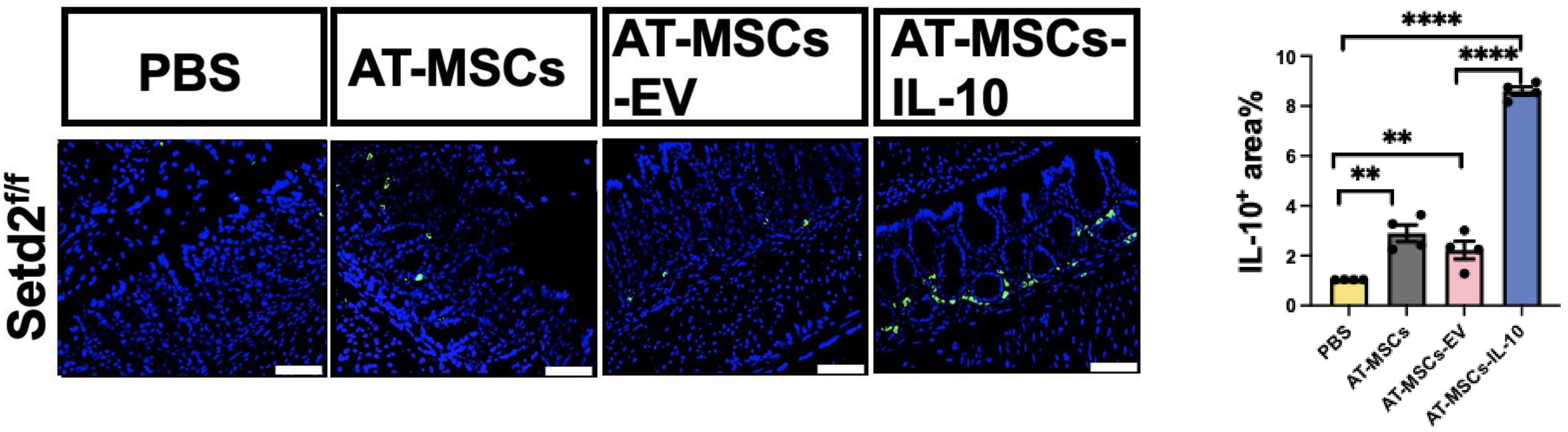

Figure S6. Targeted delivery of IL-10 by AT-MSCs upregulated the protein level of IL-10 in inflammatory colon.

(A) Immunofluorescence staining of IL-10 of colon tissues collected on day 9 from Setd2$^{f/f}$ mice were fed 2.5% DSS in drinking water and injected 1×10^6 AT-MSCs. (n = 4 per genotype). Scale Bars: 50 μm. Data represent means ± S.D., and statistical significance was determined by a student t-test. *, $p < .05$; **, $p < .01$; ***, $p < .001$.
